# Functional Connectivity Fingerprints of Emerging Reading Skill in the First Months of Schooling

**DOI:** 10.1111/desc.70142

**Published:** 2026-02-17

**Authors:** Rebecca A. Marks, Florence Bouhali, Xin Sun, Jocelyn F. Caballero, Olga Kepinska, Yuuko Uchikoshi, Adriene Beltz, Ioulia Kovelman, Fumiko Hoeft

**Affiliations:** ^1^ Department of Human Development & Family Science Purdue University West Lafayette Indiana USA; ^2^ CRPN, CNRS Aix‐Marseille University Marseille France; ^3^ Department of Language Science and Technology The Hong Kong Polytechnic University Hong Kong SAR China; ^4^ Department of Communty Health Systems University of California San Francisco California USA; ^5^ LPL, CNRS Aix‐Marseille University Aix‐en‐Provence France; ^6^ School of Education University of California Davis California USA; ^7^ Department of Psychology University of Michigan Ann Arbor Michigan USA; ^8^ Department of Psychological Sciences University of Connecticut Storrs Connecticut USA

**Keywords:** fMRI, Functional connectivity, Individual differences, Learning, Literacy, Reading development

## Abstract

The transition from pre‐reading to early word reading skill in early childhood is a time of profound developmental change. To understand changes in brain networks associated with reading development, this study examined individual differences in functional connectivity for reading at the start of formal literacy instruction. Sixty‐six kindergarteners (ages 5–6) completed a visual word processing task during functional magnetic resonance imaging (fMRI). Based on standardized literacy assessments, participants were characterized as Pre‐Readers (alphabetic knowledge but unable to read words) or Beginning Readers (some word reading ability). We compared patterns of task‐based functional connectivity between children at different stages of literacy development using confirmatory subgroup Group Iterative Multilevel Model Estimation (cs‐GIMME). cs‐GIMME is a data‐driven method that estimates individualized network connections between *a* *priori* regions of interest. Pre‐ and Beginning Readers did not differ in overall network complexity (number of functional connections between regions of interest). However, distinct periods of reading development corresponded to differences in network centrality, defined as the proportion of network connections involving specific regions of interest. Pre‐Readers had more distributed connections and greater within‐right hemisphere connectivity. In comparison, Beginning Readers demonstrated more symmetrical network organization, and greater centrality of the Visual Word Form Area and other left hemisphere language hubs. Increased reading skill was linearly associated with increased left lateralization, potentially reflecting more mature networks and greater print processing efficiency. These findings provide novel insights into child brain development during the first year of formal schooling by revealing links between emerging literacy skills and functional neural connectivity.

## Highlights

- We measured 5–6‐year‐olds’ brain activity during visual word processing and found that distinct stages of reading development were related to differences in network connectivity.
- Beginning readers had more mature networks than their pre‐reading peers.
- Overall, better readers had more left‐lateralized, focal connections between language regions.
- Results show individual differences in the reading brain in the first months of school.

## 1 Introduction

One area of debate in educational neuroscience surrounds the origins of the “reading brain.” As children learn to read, they develop functionally specialized regions that will become hubs of the reading network. This emerging specificity is driven by experience and skill development (Brem et al. 2010; Dehaene et al. 2010, Dehaene et al. 2015; Dehaene‐Lambertz et al. 2018). At the same time, there is evidence of powerful presets in the human brain that may pre‐determine how or where these specialized regions emerge (Li et al. 2020; Osher et al. 2016; Saygin et al. 2016). Put together, this evidence has driven a long‐standing theoretical question: How does reading acquisition reshape intrinsic neural organization? We offer novel insight into this question by examining 5–6‐year‐old children's functional connectivity during visual word processing to investigate whether connectivity may reflect individual differences in reading development.

### 1.1 The Emerging Reading Brain

Learning to read takes years of instruction and practice. Yet remarkably, some of the major brain changes that support fluent reading occur within the first few weeks of instruction (Brem et al. 2010, Dehaene‐Lambertz et la., 2018). One such change is the rapid emergence of the Visual Word Form Area (VWFA), a region in the visual cortex. In individuals who have never learned to read, the VWFA is equally responsive to letters as to other visual stimuli, such as faces or objects (Dehaene et al. 2015); after only weeks of kindergarten, the VWFA responds preferentially to print, and its level of activation is correlated with reading skill (Chyl et al. 2018, Chyl et al. 2023; Dehaene et al. 2010). Reading, however, is more than just seeing printed words. Readers must learn to process *language* written down, with its complex array of sounds and meanings. The change in VWFA functionality must therefore be accompanied by emerging relationships with other language hubs, allowing for coordinated brain functions to support reading. Indeed, over the first year of instruction, 6‐year‐olds show increasingly selective brain responses to print within the perisylvian language network, namely the left inferior frontal gyrus (IFG), posterior superior temporal sulcus (pSTS), and inferior parietal lobule (IPL) (Dehaene‐Lambertz et al. 2018). Yet despite the depth of knowledge around emerging specialization for reading, there remain gaps in our understanding of how these network nodes interact as children learn to read.

Evidence about functional connectivity in pre‐readers both informs and complicates our understanding of the emerging reading brain. Long before the VWFA becomes specialized for reading, its location can be predicted by pre‐existing anatomical connections (Osher et al. 2016; Saygin et al. 2016) and intrinsic resting state connectivity with the language network (Li et al. 2020). Infants demonstrate greater neural synchrony at rest between the VWFA and left language regions even within the first week of life (Li et al. 2020), mirroring patterns of resting state connectivity in adults (Stevens et al. 2017). This observation likely helps explain the remarkable speed of VWFA development during the first year of learning to read (Dehaene‐Lambertz et al. 2018). Yet, it also highlights the gaps in our knowledge regarding the mechanisms that govern the interplay between pre‐existing connections, literacy instruction, and changes in children's literacy skills. In other words, to what extent does the reading network emerge as a function of learning versus neural propensity?

This gap in our understanding may also be related to methodological differences. Inquiry into VWFA specialization has typically relied on precise localization of functional brain regions as they respond to reading tasks (Brem et al. 2010; Dehaene et al. 2010, Dehaene et al. 2015; Dehaene and Dehaene‐Lambertz 2016; Dehaene‐Lambertz et al. 2018); in other words, the location of reading‐specific brain activations. Inquiry into the pre‐existing biology of the VWFA is generally informed by studies of anatomical brain connectivity (Bouhali et al. 2014; Moulton et al. 2019; Osher et al. 2016; Saygin et al. 2016) and intrinsic connectivity of the brain at rest (Chen et al. 2019; Li et al. 2020; Stevens et al. 2017); in other words, brain connectivity that is measured when the brain is *not* reading. Other work has further revealed changes in intrinsic connectivity associated with literacy in adults (López‐Barroso et al. 2020). There is a gap between these two literatures: how do specific regions of the brain connect when an individual is actively reading?

The bridge connecting these two lines of work would examine *task‐specific* connectivity in reading development, or interactions between functional brain regions during a reading task. Yet, we know of little to no prior research that specifically examines patterns of brain connectivity *when children are looking at words* to understand task‐specific developmental change. Furthermore, foundational work on the emergence of the reading network is largely based on group averages, potentially obscuring heterogeneity and sources of individual variation. The same studies that reveal both VWFA functional specialization and intrinsic connectivity also reveal substantial variability across participants (Dehaene‐Lambertz et al. 2018; Li et al. 2020). This variability may help to inform our understanding of individual differences in learning trajectories across children, better capturing the dynamic nature of reading development (Bonte and Brem 2024).

### 1.2 Phases of Reading Development

Learning to recognize words fluently and automatically is a lengthy developmental task. Theoretical educational frameworks of word reading development distinguish between four phases of early word reading (Ehri 2005). In the *pre‐alphabetic* phase, children have limited knowledge of letters and sounds. As children gain knowledge of the alphabet, they move into the *partial alphabetic* phase, in which they may use their knowledge of letters and sound‐to‐print correspondences to recognize familiar words, but cannot decode new words. In the third phase, *full alphabetic* readers can use their knowledge of sound‐to‐print mappings to decode new words, though this process is often slow and effortful. By the time readers enter the fourth *consolidated alphabetic* phase, they have gained knowledge of larger units of print and can apply them to new words (e.g., “‐ent” in “went” and “spent”). Consolidated alphabetic readers become increasingly fluent as they encode “sight words” in memory and are able to draw on their knowledge of morpho‐syllables and spelling patterns to read novel words.

This investigation targets the transition from Pre‐Reader (*pre‐ or partial alphabetic* reading), to Beginning Reader (*full or consolidated* reading, in which children have some ability to decode novel words). The transformation from a Pre‐Reader into a Beginning Reader marks a dramatic shift, and arguably, a relatively unique moment of developmental discontinuity as readers learn to apply their basic alphabetic knowledge to recognize the linguistic building blocks that create words. Although it is well‐understood that the reading brain transforms quickly in response to literacy instruction, few studies to our knowledge have specifically investigated how neurocognitive changes map onto this specific developmental shift.

### 1.3 Connecting Brain and Behavior

The emerging reading brain “recycles,” or is built on the foundation of, existing neural systems for language and visual processing (Dehaene and Cohen 2007). The Interactive Specialization framework (Johnson 2011) further posits that early in development, cortical regions are broadly engaged in a wide range of tasks, but that over time, interactions between brain regions across tasks sharpen an individual's neural responses to become increasingly specialized and efficient.

Developmental changes within the reading network may be reflected in differences in network topology. For instance, functional connectivity during text reading revealed greater connectivity of phonology‐related regions in children, compared to greater connectivity of visual processing and semantics‐related regions in adults (Zhou et al. 2021). A separate study of connectivity during a meaning judgment task found that the middle temporal gyrus (MTG)—a key region for semantic processing (Binder et al. 2009)—emerged as a highly interconnected hub for adults but not for child readers (Liu et al. 2018). These differences in network centrality across both studies can be interpreted as evidence for increased specialization and efficiency within the adult reading network.

Another illustrative example is the change in lateralization for language and reading over the course of development. In mature readers, the reading network is typically left lateralized (Turkeltaub et al., 2003). Yet, early in reading development, children engage relatively more right hemisphere (RH) resources to support their nascent reading ability. For instance, a sample of 5‐year‐old pre‐readers demonstrated bilateral engagement of language regions when viewing letters, with similar activation magnitude in the left and right hemisphere. When children returned for a second neuroimaging session, approximately 2–3 months later, activation for letters had shifted to become left‐lateralized, more akin to adult readers (Yamada et al. 2011). A cross‐sectional study of readers ages 6–21 has additionally suggested that early RH involvement progressively decreases in relation to age and/or proficiency (Turkeltaub et al., 2003). As learners increasingly rely on the left hemisphere (LH) for reading, their functional activation in response to print becomes rapidly specialized (Dehaene‐Lambertz et al. 2018). However, it remains unknown how these specialized hubs begin to work together as a cohesive network in early reading development.

### 1.4 The Present Study

Here we addressed a question at the heart of educational and developmental neuroscience: How do differences in skill development relate to brain development and function? This study targeted 5–6‐year‐old children, all within their first months of kindergarten, but at distinct phases of learning to read. We used a precision mapping approach (Beltz and Gates 2017; Gates and Molenaar 2012) to capture individual differences in the developing reading network during a visual word processing task, and examined whether differences in network properties were related to emerging reading ability. Our inquiry aimed to advance a nuanced understanding of variability in the developing brain as it relates to literacy learning.

## 2 Method

### 2.1 Procedures

Participants completed two visits to the lab in kindergarten: first for behavioral assessments of language, literacy, and cognitive development, and second, for fMRI neuroimaging. All procedures were approved by the University of California San Francisco Institutional Review Board. Participants were selected based on the following inclusion criteria: (1) nonverbal intelligence and English vocabulary in the typical developmental range (standard scores > 80); (2) usable anatomical MRI scan and visual word matching functional data with mean framewise displacement under 5 mm; and (3) reading assessment scores that fell into the experimenter‐defined groups described below (see Participant Groups).

### 2.2 Behavioral Measures

Participants completed standardized assessments tapping into language, literacy, and cognitive skills. We assessed *nonverbal intelligence* with the Kaufman Brief Intelligence Test (KBIT‐2; Kaufman and Kaufman 2004) and *vocabulary* with the Peabody Picture Vocabulary Test (PPVT‐4; Dunn and Dunn 2007). *Reading ability* was assessed with the basic reading cluster of the Woodcock Johnson IV, using the Letter‐Word Identification subtest for *single word reading*, the Word Attack subtest for *pseudoword decoding*, and the Passage Comprehension subtest for *reading comprehension* (Schrank et al. 2014). Other *literacy‐related* assessments included *phonological awareness* (Elision subtest, CTOPP‐2; Wagner et al. 2013) and *rapid automatized naming* of numbers (Wolf and Denckla 2005).

### 2.3 Participant Groups

The present study identified participants at distinct phases of reading development (Ehri 2005, 2014) from a larger sample of 100 kindergarteners. “Pre‐Readers” had a raw score ≤ 14 on the Letter‐Word Identification subtest, and a raw score ≤ 6 on the Word Attack subtest of the Woodcock‐Johnson Tests of Achievement (Schrank et al. 2014). “Beginning Readers” had Letter‐Word Identification raw scores ≥ 20, and Word Attack raw scores ≥ 8. The final groups consisted of 34 Pre‐Readers and 32 Beginning Readers.

### 2.4 Visual Word Processing fMRI Task

During fMRI, children saw two words in sequence and determined whether the words matched (e.g., *tiger—tiger* = yes, *house—green* = no). Stimuli consisted of short, high frequency words selected from pictures of kindergarten classrooms and publicly available spelling lists to ensure their familiarity. Words were an average of 4.74 (*SD* = 1.29) letters and 3.71 (*SD* = 1.04) phonemes long. In each trial, children first saw Word 1 above a fixation cross for 2000 ms, followed by Word 2 below the fixation cross for 2000 ms, followed by a question mark for 2000 ms. The task consisted of 6 blocks of 4 trials each, separated by 12 seconds of rest. The single 3.8‐minute functional run included 12 matching and 12 non‐matching trials (see Marks et al. 2019).

### 2.5 fMRI Data Acquisition

Data were acquired using a 3T Prisma Fit MRI scanner equipped with a 64‐channel head coil. Whole‐brain functional images were acquired using a gradient‐echo echo‐planar pulse sequence: TR = 1250 ms, TE = 33.40 ms, FA = 45°, FOV = 220 mm, voxel size = 2.2mm^3^, 64 contiguous 2.20‐mm axial slices, 0‐mm inter‐slice gap. High‐resolution T1‐weighted anatomical images were collected using a matrix size of 256 × 256, 160 contiguous axial slices, voxel resolution = 1 mm, TR = 2300 ms, TE = 2.98 ms, T1 = 900  and FA = 9°.

### 2.6 fMRI Data Processing

fMRI preprocessing was carried out in FSL software version 6.0.0 (Jenkinson et al. 2012) using the fMRI Expert Analysis Tool (FEAT) with the following steps: removal of the first 11 volumes to allow for T1 equilibration effects; motion correction with MCFLIRT (Jenkinson et al. 2002); skull stripping with BET (Smith 2002); grand mean intensity normalization; spatial smoothing with a 5 mm full width half maximum (FWHM) Gaussian kernel, and B0 unwarping using Boundary‐Based Registration (Jenkinson et al. 2002; Jenkinson and Smith 2001) which also performs simultaneous registration to the high resolution T1‐image (rigid body, 6 degrees of freedom). Inclusion in the current study was limited to children with mean framewise displacement (FD) during the task < 5 mm. ICA‐AROMA (Pruim et al. 2015) was used to identify and remove motion‐related artifacts on data that passed motion quality control. As ICA‐AROMA removes a significant amount of motion‐related noise, we did not additionally regress out white matter, cerebrospinal fluid, or global signal, as these controls may introduce additional concerns (i.e., erroneous removal of effects of interest, artificial introduction of anti‐correlations) that would be difficult to identify or account for when interpreting results (Murphy and Fox 2017; Parkes et al. 2018). Denoised data were filtered with a cutoff of 0.036 Hz. In‐scanner motion did not significantly differ between Pre‐Readers and Beginning Readers (Welch's *t*(62.5) = 1.23, *p* = 0.221).

Coordinates of the left hemisphere regions of interest were derived from an independent longitudinal fMRI study of brain specialization for word reading. The regions identified by Dehaene‐Lambertz et al. (2018) are an excellent fit for the current investigation, given the similarity in sample age and schooling experience, as well as the similar demands of the single word implicit reading task used. In addition to these left hemisphere ROIs, we also extracted time series data from their right homologs (R1–R6), given prior work indicating greater bilateral engagement at the beginning of kindergarten followed by increased left lateralization (Yamada et al. 2011). Note that because the VWFA is a specific left hemisphere region, the right homolog (R6) is referred to as the right fusiform gyrus (rFG). Spheres of a 5 mm radius were defined around each of these MNI coordinates (provided in Table S1) and non‐linearly transformed to each participant's native space. Participant‐specific functional time series data were extracted from these ROIs (173 volumes each). The resultant 12 ROIs for each participant were entered into confirmatory subgroup (cs‐) GIMME analysis (see 2.7).

### 2.7 CS‐GIMME Analytic Approach

Group iterative multiple model estimation (GIMME) is a data‐driven approach that fits person‐specific sparse network models by identifying contemporaneous and lagged directed pathways between *a* *priori* ROIs. GIMME implements unified structural equation models, which combine vector autoregression and standard structural equation models; that means directional associations between variables are estimated (as in regression) for time‐lagged and time‐locked relations, respectively. GIMME begins by searching for edges (or network connections) that exist across at least 75% of participants. Specifically, the search process begins with a null model (i.e., with no associations between regions of interest), then iteratively adds whole group‐level connections (Beltz and Gates 2017). The group‐level search is guided by Lagrange multiplier tests (i.e., modification indices, or MIs) to indicate the extent to which adding a specific edge between two nodes would improve model fit for each individual, adjusting for multiple comparisons based on the number of individuals. If estimating a given connection would significantly improve model fit for at least 75% of participants, then it is estimated for everyone in the sample (Beltz and Gates 2017; Gates et al. 2014; Gates and Molenaar 2012). This process iterates, and after identifying all group‐level connections, cs‐GIMME then identifies connections that characterize the a priori subgroups. Using the group‐level connections as priors, the algorithm searches for additional edges that will improve model fit for the majority (at least 50%) of individuals in each subgroup (i.e., Pre‐readers and Beginning Readers; Henry et al. 2019). Finally, using the group‐ and subgroup‐level connections as priors, connections are added at the individual level for each participant until their person‐specific model fits the data well according to at least two standard fit indices (RMSEA ≤ 0.05, SRMR ≤ 0.05, CFI ≥ 0.95, NNFI ≥ 0.95). This person‐specific approach to connectivity mapping captures the dynamic relations between brain regions for a given individual and can detect similar patterns of connectivity across participants in the sample and in reading‐based subgroups without averaging.

In its iterative search process, the GIMME algorithm examines whether each possible path, that is, from L1 at time *t* − 1 or *t* to L2 at time t, or from L2 at time *t* − 1 or *t* to L1 at time t, may improve model fit for a given individual. Simulations show that it is important to estimate both time‐lagged (*t* − 1 to *t*) and time‐locked (*t* to *t*) relations for accurate mapping of time series data (Gates et al. 2010; Gates and Molenaar 2012). After accurately mapping all relations, we focus interpretations on contemporaneous connections which are operating synchronously and are often the focus of neuroscience explanations, including in investigations using GIMME (e.g., Goetschius et al. 2020; Becker et al. 2024).

Analyses were conducted in R version 4.3.2 with gimme 0.7–15, utilizing lavaan 0.6–17. All variables were standardized to have a mean of zero and a standard deviation of one to account for individual variability in activation changes. As described above, autoregressive coefficients were estimated to capture dependencies in the data. All analyses were conducted on contemporaneous connections. GIMME estimates the direction of associations between two nodes, and so these directions are visualized in Results; however, GIMME is not a causal model, and so, differences in directionality should be interpreted with caution (see Beltz and Gates 2017; Weigard et al. 2023).

### 2.8 Individual Metrics of Functional Connectivity

We examined three metrics to describe each child's neural network: network density, node centrality, and connection weight. Network density is the number of contemporaneous connections between nodes modeled for each participant. Node centrality is the proportion of network connections that lead to or from a specific node, or region of interest. In graph theory, centrality reflects a node's functional importance (Bullmore and Sporns 2009; Chaku and Beltz 2022), or how much this region contributes to information processing within a sparse network.

In the present study, we examine the proportion of ipsilateral connections (LH‐to‐LH and RH‐to‐RH) as compared to the proportion of contralateral connections (between LH and RH). We also examine the centrality of key reading regions, namely *centrality of the Visual Word Form Area (VWFA)*, operationalized as the number of connections leading to or from the VWFA node (L6) out of the total network density, *centrality of left STS* (L3), and *centrality of left IPL* (L4). In sparse networks, higher centrality reflects a node that plays a large relative role in network dynamics. Bonferroni correction was applied to all group comparisons.

## 3 Results

### 3.1 Behavioral Differences

Our sample consisted of 66 kindergarteners ranging from 5.1 to 6.4 years (*M*_age_ = 5.71, *SD* = 0.37). Participants were selected from a larger study of literacy development (e.g., Kepinska et al. 2023; Li et al. 2021; Marks et al. 2019) based on their reading assessment scores (Schrank et al. 2014). “Pre‐Readers” (*N* = 34) could identify letters and letter sounds but could not read words. “Beginning Readers” (*N* = 32) could identify short, high‐frequency words (e.g., *man, cup*), and decode unfamiliar nonwords (e.g., *tiff*).

Parents identified their child's ethnic and racial backgrounds as follows: 43 (65%) White or European American, 21 (32%) Asian, four (6%) Hawaiian or Pacific Islander, one Black/African American, and one American Indian/Alaska Native. Nineteen (29%) children were Hispanic or Latino/a/x. Percentages sum to more than 100, as nearly a third of parents indicated more than one ethnic or racial category. One parent indicated that their child was multiracial but did not specify further. Twelve parents (18%) indicated that their child was exposed to a language other than English beginning at birth. Languages represented included Spanish, Cantonese, Mandarin, Hebrew, and French. Mean educational attainment of parents or guardians was 4.4 on a 6‐point scale, in which 4 corresponded to a bachelor's degree and 5 corresponded to a master's.

Additional demographic and descriptive statistics of cognitive performance are listed in Table 1. On average, the Beginning Reader group was approximately 2.5 months older, and was tested approximately one month later in the school year than the Pre‐Reader group. The Beginning Reader group had higher raw performance across all literacy measures as well as measures of language and cognition; these raw differences also translated to significantly higher age‐adjusted standard scores. The groups did not differ significantly in gender composition or socioeconomic status as operationalized in terms of family income or parental education levels.

**TABLE 1:** *T*‐test comparison of demographics factors and cognitive skills between groups.

	Pre‐Readers		Beginning Readers				
Variable	*M*	*SD*	*M*	*SD*	*t*	*p*	*d*
Age	5.61	0.34	5.82	0.38	2.33	.023*	0.58
Sex	19 M / 15 F		15 M / 17 F		0.72	.472	0.18
*N* days of school at time of behavioral testing	64.44	38.88	89.34	59.06	2.01	.049*	0.50
*N* days of school at time of fMRI	91.09	47.26	112.31	63.92	1.53	.132	0.38
Nonverbal IQ (raw)	17.76	4.37	21.06	5.63	2.65	.010*	0.66
Nonverbal IQ (standard score)	103.15	11.96	110.09	15.23	2.05	.045*	0.51
Vocabulary (raw)	110.82	17.43	124.06	14.60	3.35	.001**	0.82
Vocabulary (standard score)	114.32	11.73	122.16	10.85	2.82	.006**	0.69
Letter‐Word Identification (raw)	11.24	3.06	28.59	10.44	9.05	<.001***	2.29
Letter‐Word Identification (standard score)	87.56	11.60	109.50	12.63	7.34	<.001***	1.81

*Note*: Sex coded as 1 = male, 2 = female. Standard scores have a population mean of 100 and standard deviation of 15.

### 3.2 Whole‐group Level Functional Connectivity

**FIGURE 1:**
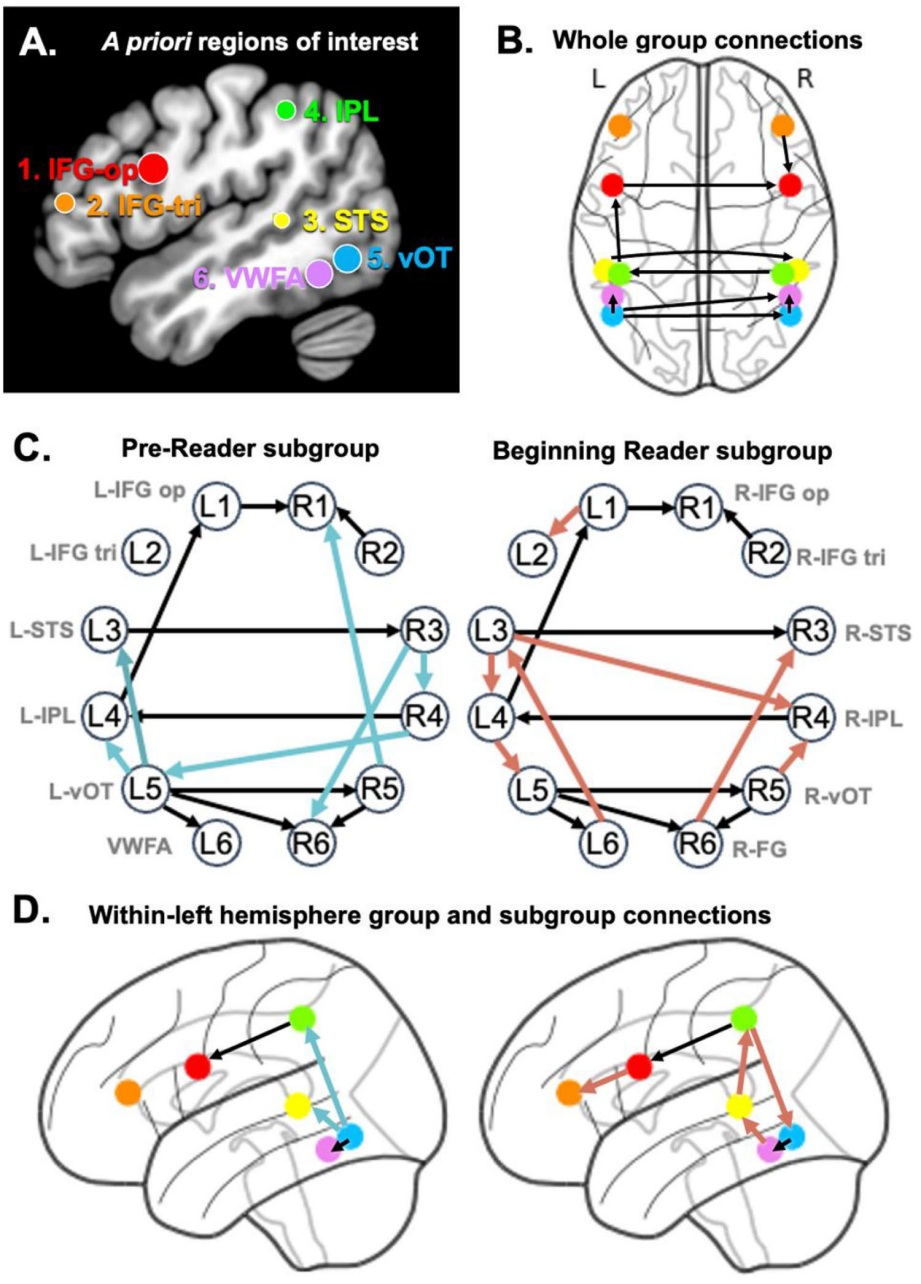
**A**. A priori left hemisphere regions of interest visualized on standard MNI template, derived from Dehaene‐Lambertz et al. (2018) coordinates. **B**. Functional connectivity at the group level determined by cs‐GIMME. **C** and **D**. Sub‐group differences in connectivity. Blue lines indicate subgroup connections among Pre‐Readers and pink lines indicate subgroup connections among Beginning Readers. Black lines reflect the whole‐group paths also visualized in B. Although GIMME estimates directed connections between nodes, these paths are contemporaneous and directionality should be considered with caution.

We investigated individual patterns of functional connectivity between *a* *priori* regions of interest of the reading network in the left hemisphere (LH) and its right‐hemispheric (RH) counterpart (Figure 1A), derived from an independent longitudinal fMRI study of brain specialization for word reading over the first year of instruction (Dehaene‐Lambertz et al. 2018). Confirmatory subgroup GIMME analysis for each individual converged normally, resulting in network models with excellent fit according to average indices.

The final models revealed nine group‐level edges that were significant for ≥ 75% of participants (Figure 1B). We observed connections between the inferior frontal gyrus (IFG) pars triangularis and inferior parietal lobe (IPL) in the left hemisphere (L4‐L1), as well as between the left ventral occipital‐temporal (vOT) region and the VWFA (L5‐L6). These network edges may reflect functional sub‐networks canonically associated with the two ‘reading routes’ for processing the phonological and lexico‐semantic aspects of written words in the dorsal and ventral streams, respectively (Hickok and Poeppel 2004; Jobard et al. 2003; Pugh et al. 2001). In the right hemisphere, there were connections within the IFG (R1‐R2), and between the vOT and the right VWFA homolog in the fusiform gyrus (FG). This connection between R5‐R6 mirrored the same associated in the left hemisphere. Group‐level edges reflected functional connectivity across hemispheres, mostly across homolog regions, including between the bilateral IFG pars opercularis (L1‐R1), STS (L3‐R3), IPL (R4‐L4), and vOT region (L5‐R5). A long‐range contralateral connection was also estimated between the left vOT and right homolog of the VWFA (L5‐R6).

### 3.3 Connectivity Differences Between Groups

The networks revealed differing patterns of connectivity between the subgroups (Figure 1C). In addition to the paths estimated at the group level, the Pre‐Reader subgroup had two additional paths between LH regions from the vOT to STS (L5‐L3) and to IPL (L5‐L4), and three additional paths between RH regions: vOT to IFG‐op (R5‐R1), and from STS to both IPL (R3‐R4) and right FG (R3‐R6). Pre‐Readers also had one long‐range, contralateral path estimated between the right IPL and left vOT (R4‐L5).

In contrast, the Beginning Reader subgroup had four additional paths between LH regions: within the left IFG (L1‐L2), from STS to IPL (L3‐L4), IPL to vOT (L4‐L5), and from the vOT back to STS (L6‐L3). Beginning Readers also had two within‐RH paths that mirrored connections estimated in the LH: from right FG to STS (R6‐R3) and between left IPL and vOT (L4‐L3). There was one contralateral connection between left STS and right IPL (L3‐R4).

**FIGURE 2:**
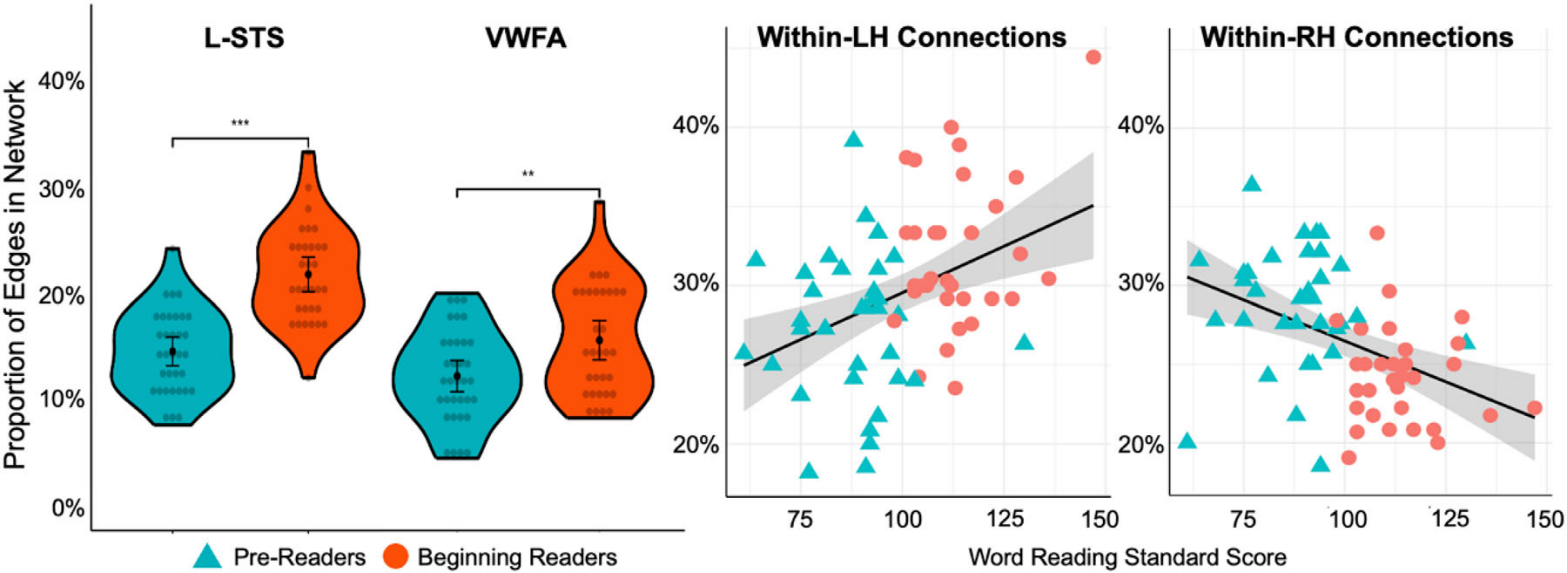
Left: Group differences in the centrality of the left STS and VWFA nodes. Right: Linear associations between the proportion of within‐left and within‐right hemisphere connections and reading scores.

We further analyzed how these connectivity patterns differed between Pre‐Readers and Beginning Readers, focusing on two aspects of network organization: density and centrality. Network density reflects the number of connections in an individual network while centrality reflects the proportion of network connections that involve a specific node; in other words, the importance of a node within the overall network. The Pre‐Reader and Beginning Reader subgroups did not differ in their network density (*t*(63.72) = 0.84, *p* = 0.567). However, they differed significantly in some measures of network organization (see Figure 2). The Beginning Reader subgroups’ network had a significantly higher proportion of within‐LH connections (*t*(63.50) = 3.92, *p* < 0.001), while the Pre‐Reader subgroup's network was characterized by a significantly higher proportion of within‐RH paths (*t*(62.62) = −5.32, *p* < 0.001). There was no group difference in the number or proportion of contralateral paths. The subgroups also differed in the centrality of three regions of interest. Beginning Readers had a higher proportion of connections emanating from and converging toward the left STS (*t*(61.15) = 6.95, *p <* 0.001), left IPL (*t*(63.41) = 2.82, *p* = 0.005), and VWFA (*t*(60.22) = 2.90, *p* = 0.004). Sensitivity analyses including both age and in‐scanner motion confirmed that all significant results were robust to covariates.

Correlation analyses revealed continuous associations between network characteristics and reading skill (Figure 2; Table 2). Partial correlations with raw scores, controlling for age, nonverbal intelligence, and in‐scanner motion revealed positive linear associations between L‐STS and L‐IPL centrality and behavioral measures of word reading, and pseudoword decoding (*r*s >0.30). STS centrality also positively correlated with phonological awareness, rapid naming, and in‐scanner task performance. VWFA centrality was correlated with stronger rapid naming and marginally associated with word and pseudoword reading (*p* = 0.063 and 0.061 respectively after Holm‐Bonferroni correction).

**TABLE 2:** Partial correlations between connectivity metrics and reading skills.

	1	2	3	4	5	6	7	8
1. L‐STS centrality	—							
2. L‐IPL centrality	.10	—						
3. VWFA centrality	.01	‐.15	—					
4. RH centrality	‐.48***	‐.27*	‐.40**	—				
5. Vocabulary	.21	.11	.16	‐.10	—			
6. Phonological awareness	.47***	.13	.15	‐.24	.45***	—		
7. Rapid naming	.39***	.17	.30*	‐.38**	.16	.37**	—	
8. Word reading	.49***	.30*	.24	‐.37**	.27*	.55***	.60***	—
9. Pseudoword decoding	.58***	.33***	.24	‐.44***	.28*	.65***	.68***	.89***

*Note: N* = 66, *p < 0.05, ** *p* < 0.01, *** *p <* 0.001 after Holm‐Bonferroni correction. Partial correlations control for age, nonverbal IQ, and in‐scanner motion. Correlations with rapid naming are calculated with the score's additive inverse so that higher scores indicate better RAN performance. STS = superior temporal sulcus; IPL = inferior parietal lobule; VWFA = Visual Word Form Area; RH = right hemisphere.

## 4 Discussion

This study addressed foundational questions on the emergence of the reading brain by examining individual differences in children's functional neural architecture for reading across distinct periods of development. Phase theories of word reading (Ehri 2005, 2014) suggest that early in literacy development, children may have limited knowledge of letters and sounds but they are unable to apply this knowledge to identify words. In later phases of reading development, children can use sound‐to‐letter mappings to decode new words, becoming increasingly fluent at recognizing units of meaning in print. At the crux of this developmental transition from being a Pre‐Reader (pre‐ or partial alphabetic phase) to a Beginning Reader (full or consolidated alphabetic phase) is a child's newfound ability to understand letters as linguistic building blocks that combine to create words. We targeted this precise developmental moment by examining person‐specific functional networks during a visual word processing task in Pre‐Readers and Beginning Readers to identify elements of continuity and discontinuity in the emergence of brain networks for reading.

Using a personalized, data‐driven approach, the present study mapped individual differences in connectivity within an *a* *priori* defined “reading network” to differences in reading skill. All participants were 5–6‐year‐olds in their first months of kindergarten but varied substantially in their reading proficiency. Individualized connectivity maps revealed that better reading was associated with a higher proportion of left lateralized connections and higher centrality of key language regions and the emerging Visual Word Form Area, likely suggesting more efficient word processing. These findings bridge evidence of intrinsic connectivity “fingerprints” that scaffold reading networks (Saygin et al. 2016) with our understanding of the rapid functional brain changes that result from learning to read (Dehaene‐Lambertz et al. 2018). Thus, precision fMRI methods provide a developmental snapshot of neurocognitive heterogeneity associated with skill acquisition.

### 4.1 Commonalities in Network Structure

Kindergartener Pre‐Readers and Beginning Readers exhibited both shared and distinct patterns of functional connectivity for visual word recognition. Both groups demonstrated connections between left and right homologs of the IFG‐opercularis, STS, IPL, and vOT nodes. This finding is consistent with prior work demonstrating bilateral engagement (Marks et al. 2019) and connectivity (Jasińska et al. 2021; Yu et al. 2018) in language regions in young children, and consistent patterns of connectivity between left and right homologs within the mature language and reading network (Sander et al. 2023). There were also network edges estimated from the bilateral vOT regions to the VWFA or right fusiform homolog mirrored across both hemispheres. This may reflect a hierarchy within the occipito‐temporal cortex. Prior research indicates that letters and letter strings are processed in a region posterior to the VWFA, whereas more anterior regions in the visual word system are sensitive to lexicality (Bouhali et al. 2014; Thesen et al. 2012; Vinckier et al. 2007). Our GIMME model estimated network edges directed from the more posterior vOT to the more anterior VWFA or rFG homolog. This is consistent with fine‐grained MEG evidence suggesting a sequential process in which activations of more posterior regions processing letters precede activations of the VWFA (Thesen et al. 2012). Group‐level network connections could lend themselves to a similar interpretation, particularly in our sample of 5–6‐year‐old kindergarteners engaged in effortful word decoding: visual information may be processed hierarchically, first as letters and letter strings, before being assembled into meaningful lexical units.

### 4.2 Differences Between Pre‐Readers and Beginning Readers

Pre‐Readers and Beginning Readers differed in network organization. Better reading skill was associated with more left‐lateralized connections, and an increasing proportion of connections between the VWFA and temporo‐parietal language regions (left STS and IPL). This discovery is aligned with the Interactive Specialization framework (Johnson 2011) which suggests that neurocognitive brain systems become increasingly specialized and efficient over time. Prior work has revealed increasingly focal patterns of brain activation for reading over development (Bitan et al. 2007; Schlaggar and McCandliss 2007; Turkeltaub et al. 2005). Extending this prior work, the higher proportion of left‐lateralized connections in Beginning Readers may reflect the increased neurocognitive efficiency associated with skill advancement. The specific connections between the left fusiform regions (vOT and VWFA) and temporoparietal language areas (STS and IPL) are also consistent with prior reading research. Better reading skill has been associated with greater connectivity between the VWFA and temporal lobe in adolescents (Aboud et al. 2016), whereas youth with dyslexia aged 9–13 demonstrate reduced VWFA‐IFG‐IPL connectivity compared to typically reading peers (van der Mark et al. 2011).

The connections estimated at the subgroup level also differed qualitatively. In particular, the Beginning Readers’ network showed more symmetry between left and right hemispheres. We observed a subgroup‐level connection between left IFG‐op and IFG‐tri, mirroring the whole group connection estimated in the RH, as well as subgroup connections within both hemispheres between VWFA‐STS and IPL‐vOT. In contrast, the subgroup level connections in the pre‐reading group were not reflected across both hemispheres. Prior research has suggested that both language and reading mechanisms may be more bilateral early in development and become left lateralized with increasing skill and experience (Olulade et al. 2020; Yamada et al. 2011). Because the present study was conducted with children in their first months of literacy instruction, findings may shed light on the developmental period at which these bilateral networks first emerge for reading. Future research with slightly more mature readers may reveal a future transition away from the right hemisphere homologs.

### 4.3 Functional Connectivity Within the Emerging Visual Word Form System

There were notable subgroup differences in connectivity between the VWFA region and the more posterior vOT region (L5, R5). Beginning Readers showed greater connectivity with the emerging VWFA than Pre‐Readers. In contrast, post‐hoc analyses revealed that Pre‐Readers had a significantly higher proportion of network connections to the left vOT (*t*(62.67) = −6.86, *p* < 0.001). This finding result may suggest less specialization within the visual word form system of Pre‐Readers. The posterior aspect of the fusiform gyrus may be involved in lower‐level processing of letter strings, but is less sensitive to lexical units (Thesen et al. 2012; Vinckier et al. 2007). Pre‐Readers may be processing the letters in the word matching task as visual forms, but not as meaningful items. In contrast, greater connectivity with the VWFA in Beginning Readers may point to emerging automaticity in word recognition. This is aligned with longitudinal evidence showing decreasing vOT activation coupled with increasing VWFA activation over the course of the first year of schooling (Dehaene‐Lambertz et al. 2018).

Notably, we did not observe VWFA‐IFG associations at the whole group or sub‐group levels. This stands in contrast to a rich body of evidence on VWFA connectivity and its relation to literacy in older children. For instance, children and adolescents who made greater gains in reading exhibited stronger fusiform to IFG connectivity during an in‐scanner reading task (Younger et al. 2017). Intrinsic VWFA‐IFG resting‐state connectivity has also been observed in infants. Neonates scanned in the first week of life showed greater connectivity at rest between hubs of the language network (i.e., Broca's area in the IFG, Wernicke's area in the STS) and the proto‐VWFA hubs than between language regions and other specialized regions of the visual system (Li et al. 2020). We observed VWFA‐STS functionality connectivity in the Beginning Reader subgroup only. The absence of the VWFA‐IFG connection in our task‐based functional connectivity maps may simply mean that kindergarteners do not yet demonstrate greater synchrony between these regions when looking at words. In other words, the pre‐wiring of the reading brain may undergo some developmental change. One possibility is that literacy instruction may “activate” this pre‐wiring, allowing new readers to build upon existing neural scaffolding to link visual representations to language and concepts. With increased specialization and skill, network connectivity will likely continue to reflect dynamic developmental change (Bonte and Brem 2024; López‐Barroso et al. 2020).

Future research that captures functional connectivity later in the kindergarten year, or once children have developed more foundational literacy skills, may reveal emerging connectivity between the VWFA and other language regions as it relates to more proficient reading. Longitudinal work may further reveal the types of reading experiences and literacy instruction that best facilitate advanced brain development for reading.

### 4.4 Limitations

The interpretation of the results from the present study is limited by its cross‐sectional design. Longitudinal research as children transition from the Pre‐Reader to Beginning Reader stage will be necessary to further pinpoint how specific skill development is associated with changes in region‐to‐region connectivity. There are also limitations of our analytic approach. Because GIMME analysis relies on a priori ROIs, there may be other important nodes or networks that might have been revealed by a whole‐brain connectivity approach. Furthermore, although the GIMME analytic framework estimates directional connections between nodes, these directions reflect statistical prediction, not a causal relation. A contemporaneous path from L‐STS to R‐STS, for instance, indicates that the activation in L‐STS is a significant statistical predictor of activation in the R‐STS at the same point in time. Reversing the direction of the path may not substantively impact model fit (Gates & Beltz, 2017). While GIMME is robust at identifying associations between pairs of nodes, and is better at identifying directionality than most functional connectivity approaches (see Mumford and Ramsey 2014), simulations indicate room for improvement in the precision and recall of connection directionality (Weigard et al. 2023). Future research may benefit from advances in network approaches to provide greater insight into causal relations between nodes.

Nevertheless, the current study sheds new light on a precise moment of developmental change in the transition to schooling. Furthermore, we offer a preliminary bridge between two sides of a long‐standing theoretical debate in educational neuroscience. One noteworthy advance is the use of task‐based functional connectivity, which reveals individual differences in brain activations specifically during visual word processing. By investigating connectivity during word reading, our findings reveal how intrinsic brain connectivity may be reshaped by skill acquisition to build efficient networks.

## 5 Conclusion

Our understanding of neuroplasticity and skill development—the idea that learning functionally “rewires” the brain—is a translational concept that has taken root across educational contexts. Here, we map individual differences in early reading ability, grounded in phase theory of reading development, onto differences in brain connectivity. Reading proficiency was associated with differences in connectivity, revealing greater centrality of left hemisphere nodes among more skilled readers. This study sheds new light on neurocognitive development for reading, revealing network heterogeneity associated with emerging reading skill in the first months of kindergarten.

## Author Contributions

RAM: conceptualization, methodology, formal analysis, writing; FB: investigation, data curation, writing (editing); XS: methodology, data curation; JFC: investigation, data curation, project administration; OK: investigation, data curation; YU: supervision, funding acquisition; AB: methodology, writing (editing), supervision; IK: writing (editing), supervision, funding acquisition; FH: supervision, funding acquisition.

## Funding

This study was funded by NIH grants R01HD078351 and R01HD096261 (to FH), R01HD111637 and R01HD092498 (to IK). RAM was supported by NIH F32HD110967. FB was supported NIH R01HD094834 (to FH), by the Institute of Convergence for Language, Communication and the Brain (ILCB, grants from France 2030 ANR‐16‐CONV‐0002 and the Excellence Initiative of Aix‐Marseille University A*MIDEX), and the French Foundation for Medical Research (Fondation pour la Recherche Médicale, FRM ARF202209015734). OK was supported by the Rubicon Fellowship (019.181SG.006) from the Nederlandse Organisatie voor Wetenschappelijk Onderzoek (NWO).

## Ethics Statement

All procedures were approved by the University of California San Francisco Institutional Review Board and were performed in accordance with the relevant guidelines and regulations.

## Conflicts of Interest

The authors have no conflicts of interests.

## Supporting information




**Supporting File 1**: desc70142‐sup‐0001‐Table.docx

## Data Availability

Subject‐level exported time series data from ROIs used for connectivity analysis is publicly available at https://osf.io/xh49r. Public archiving of anonymized functional MRI data is not permitted by the corresponding ethics approval. Preprocessed functional MRI data are available from senior author FH upon reasonable request.
