# Supplementary material for: Functional Connectivity Fingerprints of Emerging Reading Skill in the First Months of Schooling
Source: Dev Sci. 2026 Feb 17;29(2):e70142. doi: 10.1111/desc.70142 (PMC12910327; doi:10.1111/desc.70142)
Supplement: Supplementary file 1 — Supporting File 1: desc70142‐sup‐0001‐Table.docx [file DESC-29-e70142-s001.docx]

Supplementary Material for:

**Functional connectivity fingerprints of emerging reading skill in the first months of schooling**

*A priori* regions of interest were derived from an independent study of neural specialization within the reading network over the first year of schooling. 5 mm spheres were drawn around MNI coordinates reported by Dehaene-Lambertz (2018), as well as their right homologs. ROIs were warped to subject native space using a non-linear transform, and mean time series data were extracted from each ROI across the 173 timepoints.

***Table S1.*** MNI coordinates of regions of interest.

| Label | LH Node | *x* | *y* | *z* | RH Node | *x* | *y* | *z* |
| --- | --- | --- | --- | --- | --- | --- | --- | --- |
| Inferior frontal gyrus opercularis (IFGop) | L1 | -48 | 6 | 24 | R1 | 48 | 6 | 24 |
| Inferior frontal gyrus triangularis (IFGtri) | L2 | -44 | 38 | 12 | R2 | 44 | 38 | 12 |
| Superior temporal sulcus (STS) | L3 | -52 | -40 | 6 | R3 | 52 | -40 | 6 |
| Inferior parietal lobule (IPL) | L4 | -44 | -42 | 46 | R4 | 44 | -42 | 46 |
| Ventral occipital temporal region (vOT) | L5 | -48 | -64 | -8 | R5 | 48 | -64 | -8 |
| Visual Word Form Area (VWFA) | L6 | -48 | -54 | -14 | R6 | 48 | -54 | -14 |

Dehaene-Lambertz, G., Monzalvo, K., & Dehaene, S. (2018). The emergence of the visual word form: Longitudinal evolution of category-specific ventral visual areas during reading acquisition. In *PLoS Biology*, *16*(3), https://doi.org/10.1371/journal.pbio.2004103
